# The blessing of Depth Anything: An almost unsupervised approach to crop segmentation with depth-informed pseudo labeling

**DOI:** 10.1016/j.plaphe.2025.100005

**Published:** 2025-02-27

**Authors:** Songliang Cao, Binghui Xu, Wei Zhou, Letian Zhou, Jiafei Zhang, Yuhui Zheng, Weijuan Hu, Zhiguo Han, Hao Lu

**Affiliations:** a National Key Laboratory of Multispectral Information Intelligent Processing Technology, School of Artificial Intelligence and Automation, Huazhong University of Science and Technology, Wuhan, 430074, China; b PhenoTrait Technology Co., Ltd., Beijing, 100096, China; c State Key Laboratory of Plant Cell and Chromosome Engineering, Institute of Genetics and Developmental Biology, Chinese Academy of Sciences, Beijing, 100101, China; d MetaPheno Laboratory, Shanghai, 201114, China; e SpeCloud Technology Co., Ltd., Sanya, 572025, China

**Keywords:** Crop segmentation, Plant phenotyping, Depth anything, Segment anything, Efficient labeling

## Abstract

We present Depth-Informed Crop Segmentation (DepthCropSeg), an almost unsupervised crop segmentation approach without manual pixel-level annotations. Crop segmentation is a fundamental vision task in agriculture, which benefits a number of downstream applications such as crop growth monitoring and yield estimation. Over the past decade, image-based crop segmentation approaches have shifted from classic color-based paradigms to recent deep learning-based ones. The latter, however, rely heavily on large amounts of data with high-quality manual annotation such that considerable human labor and time are spent. In this work, we leverage Depth Anything V2, a vision foundation model, to produce high-quality pseudo crop masks for training segmentation models. We compile a dataset of 17,199 images from six public plant segmentation sources, generating pseudo masks from depth maps after normalization and thresholding. After a coarse-to-fine manual screening, 1378 images with reliable masks are selected. We compare four semantic segmentation models and enhance the top-performing one with depth-informed two-stage self-training and depth-informed post-processing. To evaluate the feasibility and robustness of DepthCropSeg, we benchmark the segmentation performance on 10 public crop segmentation testing sets and a self-collect dataset covering in-field, laboratory, and unmanned aerial vehicle (UAV) scenarios. Experimental results show that our DepthCropSeg approach can achieve crop segmentation performance comparable to the fully supervised model trained with manually annotated data (86.91 vs. 87.10). For the first time, we demonstrate almost unsupervised, close-to-full-supervision crop segmentation successfully.

## 1 Introduction

Crop^2^ segmentation represents a fundamental visual task in plant phenotyping. It encompasses a variety of scenarios such as field-based monitoring [1], greenhouse management [2], and large-scale agricultural surveys conducted by UAVs [3] and satellites [4]. This task also supports a multitude of agricultural practices, including the monitoring of crop growth [5], the assessment of pest conditions [6], and the estimation of yields [7]. Precise segmentation of crops can yield critical data that facilitates targeted interventions, thereby optimizing resource allocation and enhancing crop productivity [8].

Over the past decade, image-based crop segmentation approaches have shifted from classic color-based paradigms [5,[9], [10], [11], [12]] to recent deep learning-based ones [3,[13], [14], [15], [16], [17]]. Classic crop segmentation approaches have primarily relied on color-based algorithms, utilizing the distinct spectral signatures of crops in the visible light spectrum. However, these approaches often encounter challenges in complex agricultural environment, where illumination conditions, soil types, and plant varieties can vary significantly. The advent of deep learning techniques has led to substantial improvements in crop segmentation accuracy, with deep leaning models demonstrating remarkable performance in extracting relevant features from RGB images.

However, training an accurate deep crop segmentation model with strong generalization relies heavily on large amounts of data with high-quality manual annotation. The accurate annotation of data also requires considerable human labor and time, especially for segmentation-related tasks in plant phenotyping. This challenge is particularly acute in agricultural settings, where the variability in crop types, growth stages, and environmental conditions necessitates diverse and extensive datasets. Even if one can collect sufficient data, manually labeling crops in images is not only time-consuming but also prone to human errors and inconsistency, particularly when dealing with complex canopy structures [18] or overlapping plants [19].

To reduce the cost of data labeling, significant effort has been made to alleviate the labeling workload of human in crop segmentation. One straightforward approach is to decrease the number of labeled images used for training. However, given the inherent susceptibility of data labeling errors, the reduction in the amount of labeled data may introduce annotation noise, potentially leading to inaccurate feature learning. Another alternative is to replace pixel-level labeling with weaker forms of annotation, such as image-level labels or bounding box labels, a.k.a. weakly supervised learning [20,21]. Yet, these methods frequently entail intricate training procedures, which may necessitate longer training periods and greater computational resources. Self-supervised learning learns features from unlabeled data and eliminates the reliance on manual labeling [7], exploiting the latent information within unlabeled data. Nevertheless, these methods generally depend on prior knowledge or suitable self-supervised pre-tasks. In the absence of domain-specific prior knowledge, crop segmentation performance may significantly fall behind that of supervised models. Recently, some semi-automated data annotation tools based on foundation models have been developed [22,23]. They can rapidly generate pixel-level annotations and allow for manual adjustments, facilitating agricultural applications that require large-scale pseudo-labeled data. However, the quality of these annotations is not yet on par with those produced by human annotators. This is particularly challenging in high-throughput plant phenotyping, where the annotation of a crop dataset under various scenarios poses a tremendous workload. In fact, as what we will point out in this paper, label quality sometimes outweighs label quantity. Therefore, it remains an open problem on *how to reduce the labeling cost effectively for crop segmentation*.

**Fig. 1:**
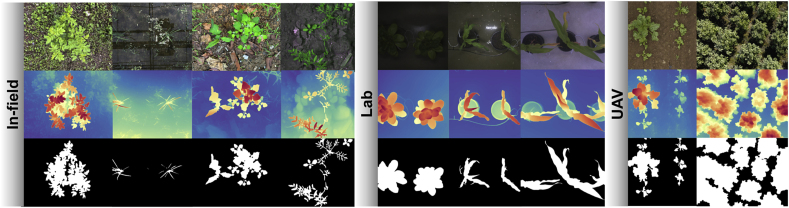
**Depth Anything model enables the extraction of high-quality pseudo crop masks from different image capturing scenarios**. From top to down, the RGB images, inferred depth maps by Depth Anything V2 [26], and pseudo crop masks extracted from the depth maps. Our work builds upon an observation that the leading monocular depth estimation model Depth Anything V2 can generate accurate depth maps robust to illumination, shadow, and cluttered background for both field crops and in-door plants. This phenomenon motivates us to extract pseudo crop masks from the depth maps and train a crop segmentation model with pseudo labels.

At this pivotal moment in the era of foundation models [24], the field of computer vision has witnessed the advent of highly adaptable and potent models, such as the Segment Anything Model (SAM) [22] and Monkey [25]. These models, pre-trained on vast amounts of data, have demonstrated exceptional performance across a wide range of visual tasks. In light of the recent advancements in vision foundation models, we observe an interesting phenomenon that the Depth Anything V2 model [26], with its sensitivity and accuracy in capturing subtle depth boundaries, is particularly adept at delineating crop regions and its boundary details in complex scenarios, especially when there exhibits an obvious depth contrast between crops and the background (Fig. 1). This observation has immediately motivated us to investigate the potential of this model in generating pseudo crop masks for training crop segmentation models, with the goal of reducing the necessity for manual annotation.

To implement the idea above, the first and the most important thing is *how to generate reliable pseudo labels of a sufficient number of training data*. Since the depth map is encoded as a single-channel gray-scale image, classic gray-scale image thresholding approaches could be adopted to extract the crop mask. However, due to the bias of shooting angle, many field images cannot produce consistent background depth such that classic image thresholding cannot work. To address this, we propose gradient-guided histogram thresholding to overcome the bias of background depth. Specifically, we propose to normalize and modulate the depth map with gradient and edge information to highlight crop regions. In this way, a sufficiently large candidate training image pool can be guaranteed. Then, by introducing fast coarse-to-fine manual screening by naked eyes, low-quality and obviously erroneous masks would be filtered out at a low cost, leading to a high-quality pseudo labeled crop segmentation training set. In addition, we also introduce a two-stage self-training strategy to further condense the training labels and apply depth-informed edge-preserving filtering as post-processing to remove noise.

To validate the generality of DepthCropSeg to base crop segmentation models, we evaluate four semantic segmentation models including U-Net [27], DeepLabV3+ [28], SegFormer [29], and Mask2Former [30], and we also consider two segmentation foundation models SAM [22] and HQ-SAM [31] as comparing baselines due to their class-agnostic segmentation ability. Experimental results on ten public crop segmentation datasets and on self-collected data show that DepthCropSeg is able to achieve surprisingly good crop segmentation performance. Compared with masks directly generated from depth maps and segmentation foundation models, DepthCropSeg shows significant improvements, approaching the level of performance with careful manual labeling. We also conduct extensive ablation studies to validate each contribution of our strategies, designs, and technical improvements.

The main contributions of this work include the following:

• We show that the Depth Anything model can help generate high-quality pseudo crop segmentation masks, thus evading the need of manual pixel-level annotations;
• We introduce DepthCropSeg, a depth-informed crop segmentation approach featured by depth-informed pseudo mask labeling, depth-informed two-stage self-training, and depth-informed post-processing, which enables almost unsupervised crop segmentation.
• We benchmark the segmentation performance of DepthCropSeg on 10 public crop datasets including field-based, laboratory, and UAV scenarios; To the best of our knowledge, it is the first time that close-to-full-supervision performance is achieved in an almost unsupervised way.

## 2 Related work

We review work related to crop segmentation models and crop segmentation with less annotations.

### 2.1 Crop segmentation approaches

Crop segmentation approaches can be categorized into classic crop segmentation and deep crop segmentation.

**Classic Crop Segmentation** This line of approaches mainly include color-based [5,32], region-based [10,11], and clustering-based ones [33]. They usually rely on color, texture, and shape features to identify and segment crop regions. Yu et al. [5] proposes the AP-HI segmentation model that exploits the relation between hue and intensity to segment maize plants. Ye et al. [32] further addresses the segmentation of highlight regions with graph-based Markov random fields. Lu et al. [10] builds a region-based color model for simultaneous crop and tassel segmentation. A similar idea is also followed by Ref. [11] to tackle the segmentation of in-field cotton. While these approaches does not need extensive training or data, they often require careful parameter tuning to achieve satisfactory segmentation results.

**Deep Crop Segmentation** To enhance the performance of classic crop segmentation approaches, deep learning based ones are introduced. They require less human intervention but exhibit stronger performance and adaptability. Deep crop segmentation models typically follow the fully convolutional architecture such as Fully Convolution Network (FCN) [34], U-Net [27], and DeepLabV3+ [28]. Lottes et al. [13] proposes a sequential FCN that exploits spatial information of plant arrangement from image sequences to improve crop-weed segmentation in precision agriculture. Combining a threshold-based approach and a modified U-Net architecture, Zou et al. [3] demonstrates the effectiveness of two-step segmentation on weed density evaluation. Based on high-resolution remote sensing images, Du et al. [14] exploits DeepLabV3+ to extract crop regions. Recently, Transformer architectures such as SegFormer [29] and Mask2Former [30] have also been introduced to address crop segmentation. Gao et al. [15] proposes a self-supervised semantic segmentation approach on green fraction estimation in rice and wheat crops, where the SegFormer achieves the best results on the Sim2Real dataset. Darbyshire et al. [16] presents Hierarchical Mask2Former, a model that adapts state-of-the-art panoptic segmentation for precision agriculture, enabling simultaneous crop, weed, and leaf identification with efficient architecture. The approaches above, however, require manually labeled data of large quantity and high quality at the pixel level, which is often time-consuming and labor-intensive.

**Remark** Our work follows the deep crop segmentation paradigm considering its superior performance in challenging scenarios. Compared with the fully supervised training employed in deep crop segmentation, we show that we can train a crop segmentation model without the need of pixel-level manual annotation.

### 2.2 Crop segmentation with less manual annotations

The training of deep crop segmentation models requires extensive pixel-level manual annotation as data labels. To reduce reliance on manual annotations, researchers have explored various approaches in recent years such as weakly supervised learning, self-supervised learning, and transfer learning. Some semi-automated data annotation tools also emerge.

**Weakly Supervised Crop Segmentation** leverages partially labeled data or weaker labels, combined with unlabeled data, to reduce the need for precise pixel-level annotations of crop masks. For instance, Milioto et al. [20] proposes a weakly supervised approach to achieve real-time semantic segmentation of crops and weeds in agricultural fields. Wu et al. [21] proposes a method for crop organ segmentation and disease recognition based on weakly supervised deep convolutional neural network (DCNN). Only the bounding box is required.

**Self-Supervised Crop Segmentation** extracts features from unlabeled data and applies these features to crop segmentation. Saeed et al. [7] presents a DNN approach to accurately predict crop yields. By employing a guided feature selection technique, the model automatically identifies and prioritizes the most critical genotype and environmental variables, enabling crop segmentation with reduced reliance on manual annotations. Gao et al. [35] develops a self-supervised training pipeline conditioned on simulated data.

**Transfer Learning Based Crop Segmentation** applies a model trained on one domain to another domain, thereby reducing the need for new labeled data. Bosilj et al. [36] uses an encoder-decoder CNN and three datasets with different crop types to efficiently transfer the knowledge from one crop type to another, which reduces the training time for up to 80 ​%. Yang et al. [37] presents a two-step transfer learning approach using the synthetic in-vitro soybean pods dataset. They successfully transfer from simulation to reality, as well as from in-vitro segmentation to on-branch segmentation.

**Semi-Automated Crop Annotation** has gained much attraction recently. An increasing number of researchers have employed the Segment Anything Model (SAM) to achieve semi-automated annotation without the necessity for further training. SAM has been trained using over one billion masks from 11 million images [22]. It exhibits high-quality class-agnostic segmentation comparable to fully-supervised models. In particular, Lu et al. [23] adopts a SAM-based semi-auto-labeling tool LabelMe to reduce the labeling cost in maize plant detection.

**Remark** While much progress has been made in reducing the reliance on manual annotations, there has been limited comparison of these methods with manually labeled data in terms of performance. Our work introduces the use of depth information to acquire segmentation masks on RGB images and benchmarks the performance of a number of approaches, providing a fresh insight to this task.

## 3 Materials and methods

In this section, we first describe how we organize the public datasets for crop segmentation and then define the problem, compare different mask generation ideas, and present our Depth-Informed Crop Segmentation (DepthCropSeg) solution.

### 3.1 Crop segmentation data

For ease of quantitative validation, we mainly leverage public crop segmentation datasets due to available labeled crop masks. The availability of the training crop masks also allows us to set a fully supervised baseline as the upper bound of pseudo labeling. In addition, we also consider a portion of data captured by smartphones and by a commercial high-throughput plant platform deployed in real business scenarios, in order to validate the generalization ability of the model.

#### 3.1.1 Public crop segmentation datasets

**Fig. 2:**
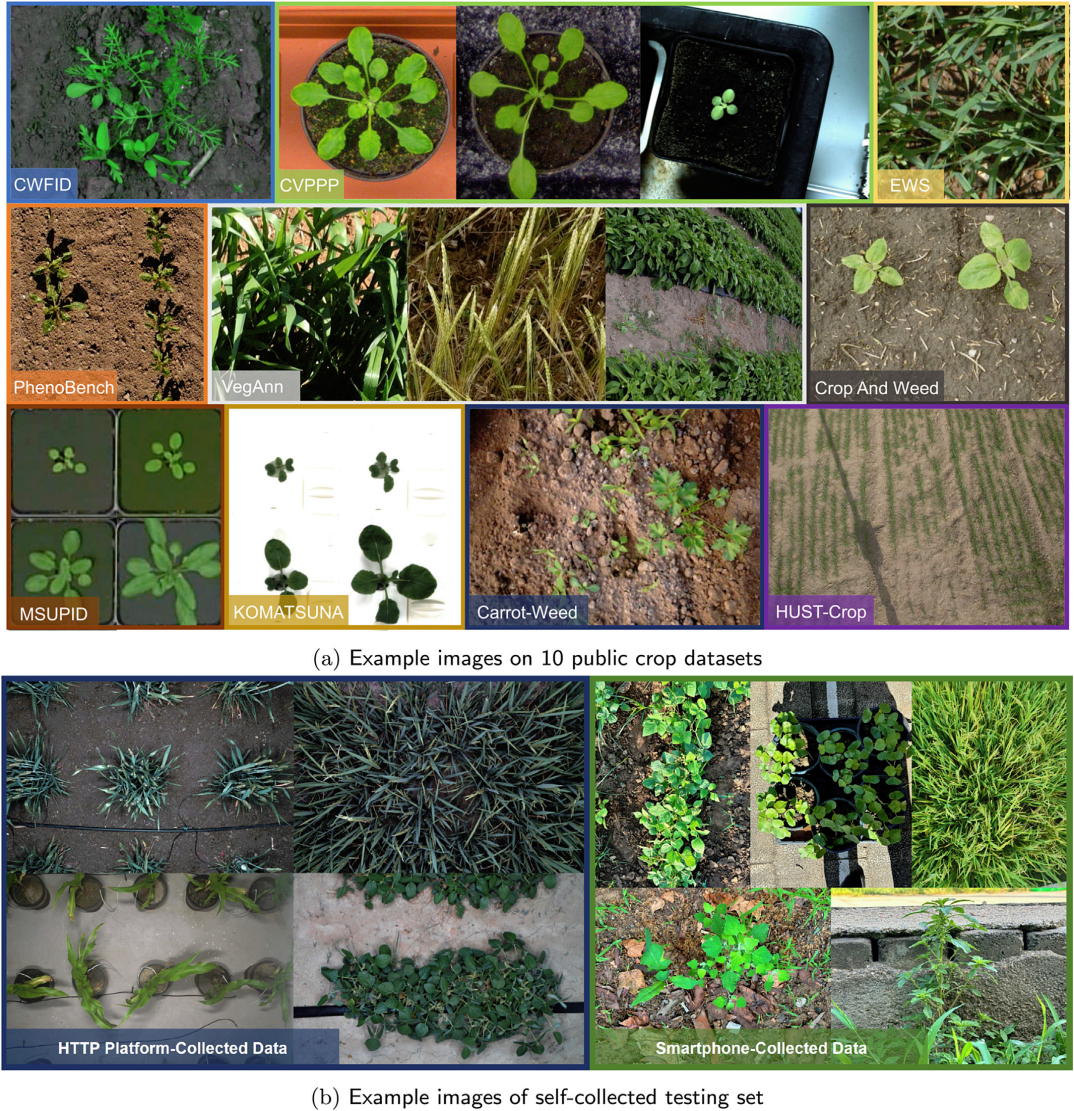
**Examples images of collected crop datasets**. Images shown in (a) are from the 10 public crop datasets, which are captured under in-field, laboratory, and UAV scenarios. Images shown in (b) are self-collected testing set. They were gathered using a high-throughput plant phenotyping platform and smartphone.

We leverage 10 publicly available plant datasets covering field-based, indoor, and UAV scenarios. Example images are shown in Fig. 2(a), these data sets cover different plant varieties, and images are captured at different imaging platforms from distinct imaging views. Characteristics of different plant segmentation datasets are shown in Table 1. Our training set consists of 17, 199 images from the first 6 datasets, and the testing set gathers all the testing sets from the 10 datasets, with 5998 images in all. Note that, we preprocess crop and weed labels and consider them both as the foreground crop category. A brief introduction of each plant dataset is introduced in what follows:

• The CWFID dataset [38] comprises 60 images captured at a commercial organic carrot farm in Northern Germany using a top-down camera system on the Bonirob robot. Images were acquired in both indoor and outdoor environments under controlled illumination conditions. Among the 60 images, 20 are used for training, and 40 for testing. Annotations are provided in the form of vegetation masks and manual crop/weed labeling. The images have a resolution of 1296 ​× ​966 pixels.
• The CVPPP dataset [39] consists of raw and annotated top-view color images of Arabidopsis and tobacco plants, captured in controlled indoor environments under consistent illumination conditions. Each image is associated by expert annotations and metadata, detailing plant properties and experimental conditions. These images are collected using different camera setups, with resolutions ranging from 5 to 7 megapixels. 501 images are used for testing.
• The EWS dataset [40] comprises 190 manually selected images of different winter wheat genotypes. The images, taken from 2017 to 2020, are split into 154 for training and 36 for testing. The annotations include manually labeled binary masks for plants and soil, with images cropped to 350 ​× ​350 pixels.
• The PhenoBench dataset [41] has high-resolution images of sugar beet fields captured by UAVs under natural illumination conditions. The images are taken over multiple days at different growth stages of the plants, resulting in a resolution of 1, 024 ​× ​1, 024 pixels. The dataset includes 2, 872 images, with 772 images used for testing. Annotations are provided at both the plant and leaf levels.
• The VegAnn dataset [42] includes 3, 775 multi-crop RGB images obtained under diverse illumination conditions through various systems and platforms. These images are taken from multiple locations and across different phenological stages of crops, with a resolution of 512 ​× ​512 pixels. The dataset is split into five distinct training, validation, and test sets, with annotations provided for vegetation and background segmentation at the pixel level.
• The Crop And Weed dataset [43] has a set of 74 plant classes, in which 16 representing crop species, and the remaining 58 are considered as weeds. The dataset is divided into experimental and application sets, with a total of 4, 990 images for training and 3, 044 images for testing. Annotations include labeled bounding boxes, semantic masks, and stem positions. The images are captured at a resolution of 1, 920 ​× ​1, 088 pixels.
• The MSUPID dataset [44] consists of two varieties of plants (Arabidopsis and beans), captured by four types of imaging sensors: fluorescence, infrared, RGB color, and depth imagery. The dataset comprises 2, 160 images for Arabidopsis and 325 images for bean, with manual annotations provided for leaf identification, leaf tips, and leaf segments. The Arabidopsis images are captured at a resolution of approximately 240 ​× ​240 pixels for fluorescence and infrared, and the bean images have a resolution of 1, 000 ​× ​640 pixels.
• The KOMATSUNA dataset [45] includes RGB-D and multiview images of Komatsuna in early growth stages. Images are captured every 4 ​h over 10 days, with 166 ​× ​190 pixels for plant images and 640 ​× ​480 pixels for the RGB-D setup. The dataset includes manual pixel-level leaf annotations, and the images are used for both spatial and temporal analysis of plant growth.
• The Carrot-Weed dataset [46] comprises 39 high-resolution RGB images of young carrot seedlings. The images are annotated with pixel-level masks distinguishing between carrot plants, weeds, and ground. Each image has a resolution of 3264 ​× ​2448 pixels.
• The HUST-Crop dataset [47] has 340 high-resolution RGB images of four crop varieties (cotton, maize, rice, and wheat) captured under various environmental settings, including sunny, cloudy, and rainy conditions. The images are collected from a 5-m-height pole, with 261 images under near-ground conditions and with 79 under canopy-level ones. The images are manually annotated using Adobe Photoshop CS5, and detailed ground-truth segmentation masks are included for each crop.

**Table 1:** Characteristics of different crop segmentation datasets.

Dataset	Years	Varieties	Resolution	Imaging platform	Training set	Testing set
CWFID	2015	Carrot/weed	1296 ​× ​966	In-field	✓	✓
CVPPP	2017	Mixed	Mixed	Lab	✓	✓
EWS	2022	Wheat	350 ​× ​350	In-field	✓	✓
PhenoBench	2023	Mixed	1024 ​× ​1024	UAV	✓	✓
VegAnn	2023	Mixed	512 ​× ​512	In-field	✓	✓
Crop And Weed	2023	Mixed	1920 ​× ​1088	In-field	✓	✓
MSUPID	2015	Mixed	Mixed	Lab		✓
KOMATSUNA	2017	Komatsuna	480 ​× ​480	Lab		✓
Carrot-Weed	2017	Carrot/weed	3264 ​× ​2448	In-field		✓
HUST-Crop	2020	Mixed	Mixed	In-field		✓
Total #Images					17,199	5998

#### 3.1.2 Additional self-collected testing data

To further ascertain the adaptability and precision of our segmentation approach across diverse real-world agricultural settings, we have curated an additional testing set (Fig. 2(b)). This testing set consists of two distinct subsets; each offering a unique insight into the model performance under varied conditions, including:

• HTTP Platform-Collected Data: This subset was collected using a state-of-the-art high-throughput plant phenotyping (HTTP) platform TraitDiscover (PhenoTrait Technology Co., Ltd.) located in various regions across China, including four research stations of Chinese Academy of Sciences (CAS) and the MetaPheno laboratory, Shanghai. The four research stations of CAS are: i) the Changping research station of Institute of Genetics and Developmental Biology, ii) the Liaoheyuan research station of The Northeast Institute of Geography and Agroecology, iii) the Chengdu Plain Agricultural Ecology Research Station, and iv) the research station of Xishuangbanna Tropical Botanical Garden. The platform provides a controlled and systematic view of plant growth and allows for the capture of high-resolution images under varying conditions. The images, totaling to 176 images, encompass a wide range of plant species and developmental stages. Each image is captured at a resolution of 5472 ​× ​3648 pixels, offering detailed visual information necessary for accurate segmentation tasks.
• Smartphone-Collected Data: This subset consists of images captured using smartphones by field agronomists and researchers. This collection process introduces a level of variability in image quality, illumination conditions, and angles that is reflective of real-world usage scenarios. This subset comprises 52 images with varied resolution, featuring a variety of plant species commonly found in agricultural settings. This part of the dataset is crucial for assessing the robustness of the model in non-ideal imaging conditions and its practical utility in agricultural field settings.

### 3.2 Problem formulation

Given an RGB crop image ***X*** and an inferred depth image ***D*** from any monocular depth estimation model D conditioned on ***X***, our goal is to train a crop segmentation model F to generate the crop mask $\hat{M}$ from ***X*** to be as close as possible to the ground-truth crop mask ***M***_*gt*_, without any pixel-level manual annotations. To produce the supervision signal, the pseudo mask ***M*** is generated from ***D*** following a certain mask generation function G. The whole problem formulation takes the form

(1) $$\left\{\begin{array}{l} M=G\left[D\left(X\right)\right]\;\\ F^{*}\leftarrow optim\left(F|X,M\right)\;\\ \hat{M}=F^{*}\left(X\right)\; \end{array}\right).$$

### 3.3 Depth Anything Model revisited

**Fig. 3:**
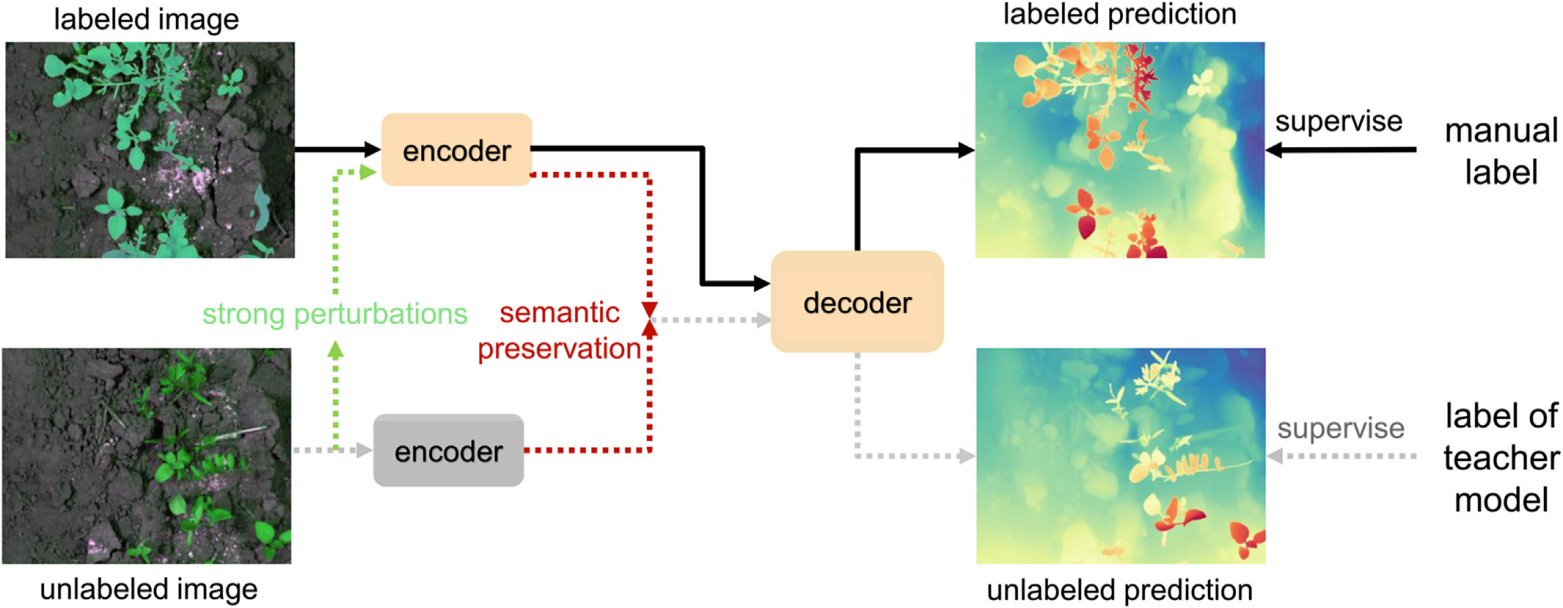
**Part of training pipeline of the Depth Anything model**. The training of the student model based on the encoder-decoder structure is divided into two routes, one under the supervision of labeled data and one based on unlabeled data that has been strongly perturbed and semantically preserved, and under the supervision of pseudo-labels generated by the teacher model.

In this work, we adopt the improved version Depth Anything V2 [26] to acquire the depth image ***D***. Depth Anything V2 builds upon its initial version Depth Anything V1 [48]. Here we first revisit the principle of the V1 Model. In monocular depth estimation, to solve the difficulty of building a large-scale data set with tens of millions of depth labels, the Depth Anything Model (DAM) is proposed [48]. In particular, DAM exploits information from unlabeled images by first training a supervised encoder-decoder teacher model. The teacher model is then used to generate pseudo-labels on unlabeled data. Finally a student model is trained with both the labeled and pseudo-labeled data, as shown in Fig. 3. During the student training, strong perturbations are applied to the unlabeled data to improve the generalization ability, and the semantic information from the labeled data is also used by semantic preservation for better supervision.

**Fig. 4:**
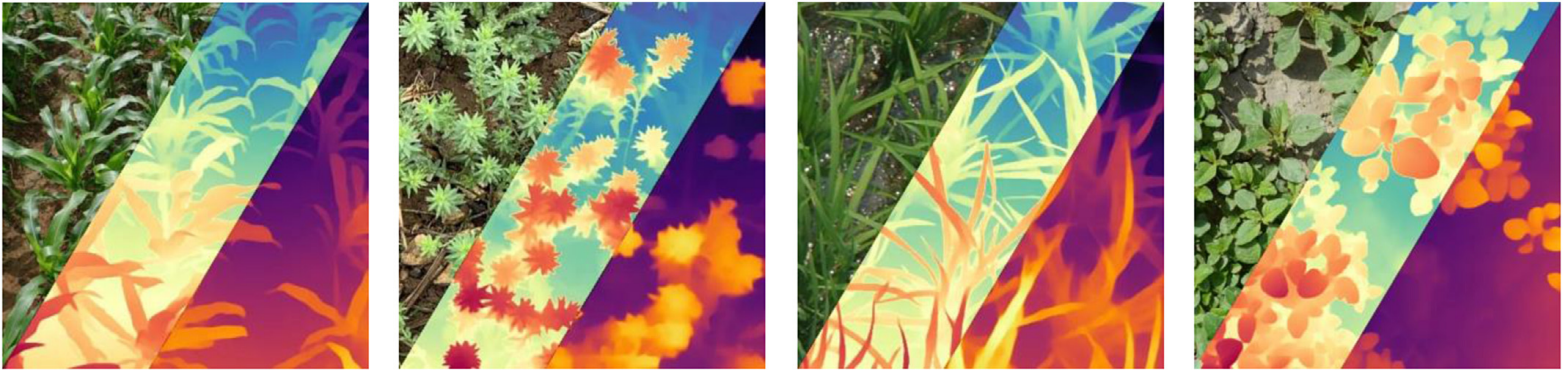
**Comparison of Depth Anything V1 and Depth Anything V2**. For each image, from left to right, the original RGB image, the depth map of V2, and the depth map of V1.

Compared with V1, V2 replaces real training images with synthetic ones, expands the capacity of teacher model, and teaches the student model via the bridge of large-scale pseudo-labeled real images. As a result, it shows better robustness and extracts fine-grained details. The debate between using real and using synthetic data for training has lasted for a long time. Real labeled images, despite being realistic, have label noise and omission of details. In contrast, while synthetic images can easily have all fine details being correctly labeled, there remains two limitations of synthetic images that prevent its real-world generalization: i) synthetic images are rather clean and organized, but real images contain more randomness; and ii) the scene coverage of synthetic images is limited. To build on the strengths and avoid the weaknesses of synthetic images, the teacher model of V2 is based on the DINOv2-G encoder [49], trained on high-quality synthetic images, and then assigns pseudo-depth labels on unlabeled real images. Finally, the student model is trained using only exact pseudo-labeled images for robust generalization. As shown in Fig. 4, V2 is more accurate in labeling fine details and more suitable for plant phenotyping because plants often exhibit fine details.

This model allows us to exploit a large amount of unlabeled plant data and to generate pseudo pixel-level labels, which greatly reduces the difficulty of constructing a manually labeled segmentation dataset.

### 3.4 Depth-informed pseudo mask labeling

In plant phenotyping, crop photos are commonly taken from a top-down view. Under this practice, crops often appear at a distinct depth plane compared with the relatively flattened ground such that crops can have larger values in the inferred depth map. Concurrently, the depth boundaries between crops and ground naturally form a significant gradient due to the disparity in elevation, which makes it easy to separate foreground crops. Based on these priors and the depth map is in fact represented as a single-channel gray-scale image, classic gray-scale image-based thresholding segmentation methods can be applied to extract coarse crop masks. In this work, we first study two representative image thresholding approaches and then propose a new depth-informed thresholding one for pseudo mask generation.

#### 3.4.1 Mask labeling with OTSU thresholding

The OTSU thresholding, developed by Nobuyuki Otsu in 1979 [50], is an automatic image thresholding method for binarizing grayscale images. The idea of OTSU thresholding is to iterate over all pixel values to minimize the inter-class variance. Variance is a measure of the uniformity of the gray scale distribution. Ideally, a good image thresholding implies large foreground-background inter-class variance $\sigma_{B}^{2}$ and small same-class intra-class variance $\sigma_{W}^{2}$. Therefore, the segmentation that maximizes the inter-class variance is preferred, which takes the form

(2) $$\arg\;\underset{T}{\max}\sigma_{B}^{2}\left(T\right),$$

where $\sigma_{B}^{2}\left(T\right)=w_{0}\left(T\right)w_{1}\left(T\right)\left(u_{0}\left(T\right)-u_{1}\left(T\right)\right)$, *w*_0_(*T*) and *w*_1_(*T*) denote the probabilities of the occurrence of background and foreground pixels at the intensity *T*, respectively, and *u*_0_(*T*) and *u*_1_(*T*) are the mean values accordingly. More detailed derivations can be found in Ref. [50]. Given the depth image ***D***, the pseudo mask ***M*** can be obtained by searching the optimal *T*∗ under OTSU criterion, *i.e.*,

(3) $$M={otsu\text{\_}threshold}_{T^{*}}\left(D\right).$$

#### 3.4.2 Mask labeling with generalized histogram thresholding

The generalized histogram thresholding (GHT) [51] is a histogram-based image threshold approach. GHT generalizes the idea of Otsu image thresholding, minimum error thresholding, and weighted percentile thresholding so that it allows for successive interpolation between these three algorithms via four different parameters that control the importance of specific probability distributions such as Gaussian Mixtures, inverse Chi-squared, and *β* distributions. This approach assumes that each pixel of the input image is generated by mixing two probability distributions, and the optimal objective is to maximize the joint probability of all pixels. In this way, a GHT criterion is defined by

(4) $$\arg\;\underset{T}{\max}\left(f^{\left(0\right)}+f^{\left(1\right)}\right),$$

where *f*^(*k*)^ denote the log-likehood of the data at each split for each Gaussian. The specific form for *f*^(*k*)^ is determined by the four parameters. More technical details can be found in Ref. [51]. Given the depth image ***D***, the pseudo mask ***M*** can be obtained by searching the optimal *T*∗ under GHT criterion, *i.e.*,

(5) $$M={GHT}_{T^{*}}\left(D,\nu ,\tau ,k,w\right),$$

where *ν*, *τ*, *k*, and *w* are the four distribution-related hyper-parameters. We follow the default hyper-parameters provided by the authors.

#### 3.4.3 Mask labeling with proposed gradient-guided histogram thresholding

**Fig. 5:**
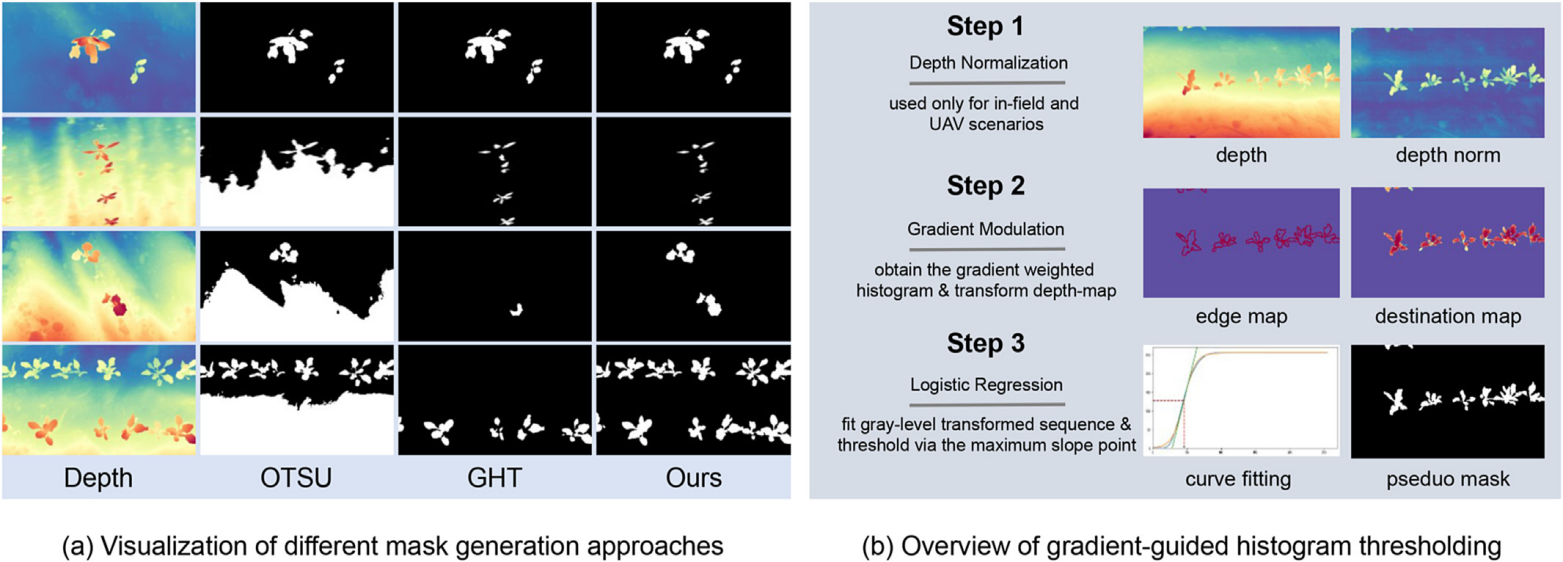
**(a) Comparison of different approaches for pseudo mask generation and (b) the overview of proposed pseudo mask generation**.

When generating pseudo masks from depth maps, we find that the depth values of background in many in-field images show a significant drop-off due to shooting angle and model bias. The two thresholding approaches above do not perform well with biased background. As shown in Fig. 5(a), the OTSU-thresholding and GHT approach usually segment the background into foreground. To obtain higher-quality pseudo masks, we propose the gradient-guided histogram thresholding.

For gray-scale maps converted from RGB images, the gradient information comes primarily from color differences and texture properties. However, in ideal crop depth maps, significant pixel gradients are mainly derived from the depth difference between ground and crops. This inspires us to introduce gradient and edge information into classic thresholding.

To overcome the biased background in estimated depth images, we first perform depth normalization to rescale the biased background depth values. Concretely, given the inferred depth image ***D***, we subtract each row and column with its row and column minimum, by order, defined by

(6) $$\left\{\begin{array}{l} D_{n}\left(i,j\right)=D\left(i,j\right)-min\left(D\left(i,:\right)\right)\;\\ D_{n}\left(i,j\right)=D_{n}\left(i,j\right)-min\left(D\left(:,j\right)\right)\; \end{array}\right).$$

**Fig. 6:**
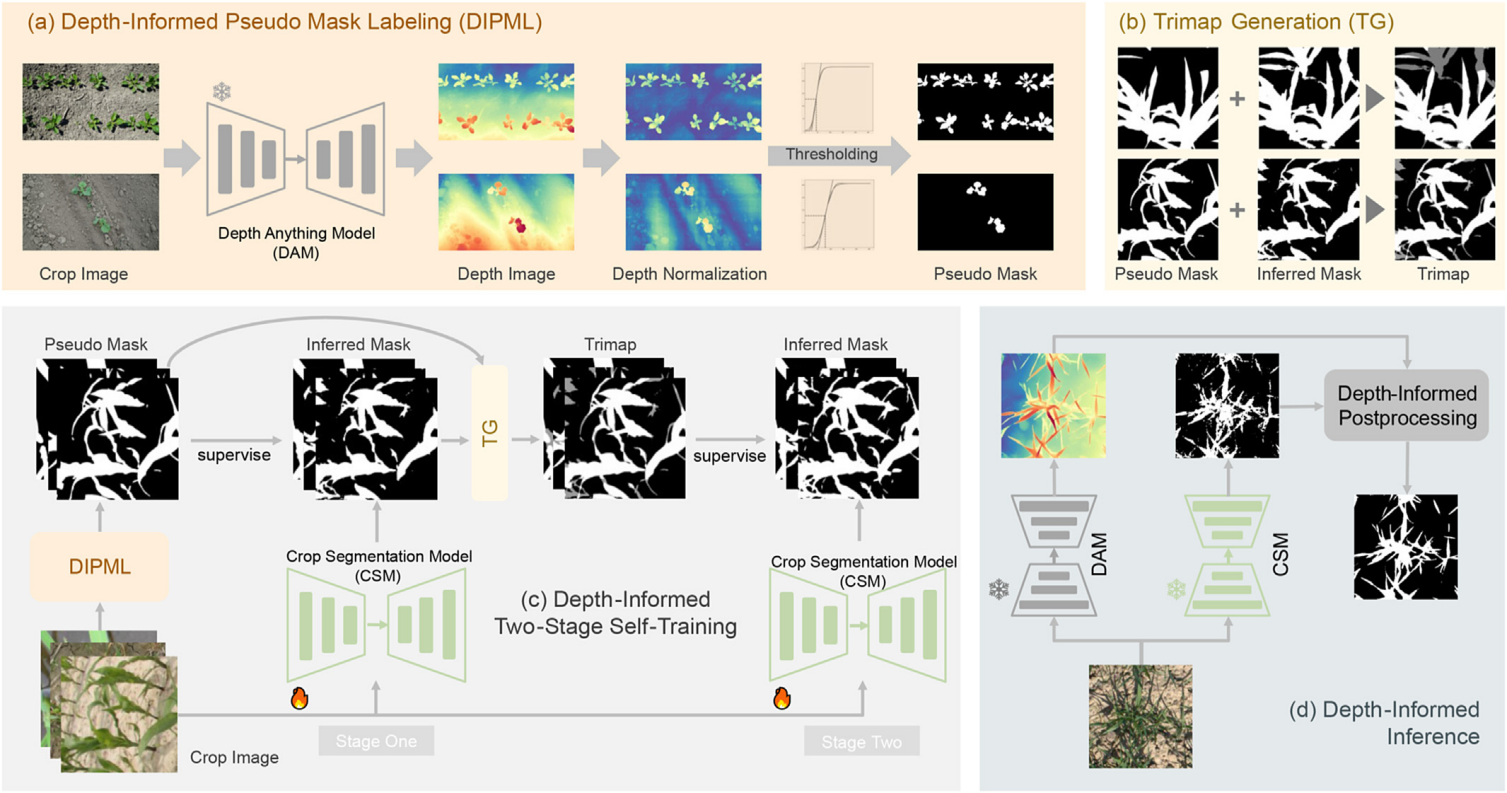
**Technical pipeline of DepthCropSeg**. From (c) the depth-informed two-stage self-training, DepthCropSeg enables almost unsupervised training pipeline with two key modules. In (a) depth-informed pseudo mask labeling, pseudo masks are generated by depth-image-thresholding, which are the labels in the first-stage training. In the second-stage training, through (b) trimap generation, confident region are extracted as the refined label. During the (d) depth-informed inference, DepthCropSeg improves the quality of inferred-mask via depth-informed postprocessing.

The effect of depth normalization can refer to Fig. 6(a). Note that we only apply this step to in-field and UAV scenarios. To further reduce background interference, we introduce the gradient and edge information. In particular, we use the Sobel operator to obtain the gradient map ***G*** ​= ​{*g*_*ij*_} and use the Canny operator to compute the edge map ***E*** ​= ​{*e*_*ij*_} [52] from ***D***_*n*_ where *g*_*ij*_ and *e*_*ij*_ are the (*i*, *j*)-th element of ***G*** and ***E***, respectively. With the edge map ***E***, we slightly dilate ***E*** to connect some noncontinuous edges and generate the coarse target mask ***M***_*t*_ via finding contours and filling the internal regions. To suppress the background noise effectively, we set the gradient value into *p* levels and obtain the gradient-level map $L=\left\{l_{ij}\right\}=\left\lfloor \frac{p\cdot g_{ij}}{\max\left(G\right)}\right\rfloor$. The weight for the *k*-th gradient level can be computed by

(7) $$\gamma_{k}=\frac{\sum_{l_{ij}=k}e_{ij}}{\sum_{l_{ij}}e_{ij}},\;0\leq k\leq p-1.$$

We then normalize the gray level sequence $R\left(T\right)=\sum_{d_{ij}=T}\gamma_{l_{ij}}e_{ij}$ to obtain the normalized sequence $S\left(T\right)=\frac{255}{\sum_{k=0}^{255}R\left(k\right)}\cdot \sum_{k=0}^{T}R\left(k\right)$. The final transformed depth map ***D***_*t*_ takes the form ***D***_*t*_ ​= ​*S*(***D***)_*n*_ ∩***M***_*t*_. To obtain the segmentation threshold for ***D***_*t*_, we fit the normalized transformed sequence *S*(*T*) with a parametric sigmoid function

(8) $$f_{sigmoid}=\frac{c}{1+\exp\left(-a\cdot \left(n-b\right)\right)},$$

where *c* is a constant of 255, the range of *b* is from 0 to 255, and the value of *a* can be set from 0 to 5. Both *a* and *b* are learned by fitting *S*(*T*). Finally, we select the point w.r.t. the slope maximum in the fitted curve as the threshold to binarize the transformed depth map ***D***_*t*_ to obtain the pseudo mask ***M***. The overview of this approach refers to Fig. 5(b).

### 3.5 Depth-informed crop segmentation

In this section, we elaborate our approach on crop segmentation in terms of training sample selection, two-stage self-training, and post-processing.

#### 3.5.1 Sample selection with coarse-to-fine manual screening

**Fig. 7:**
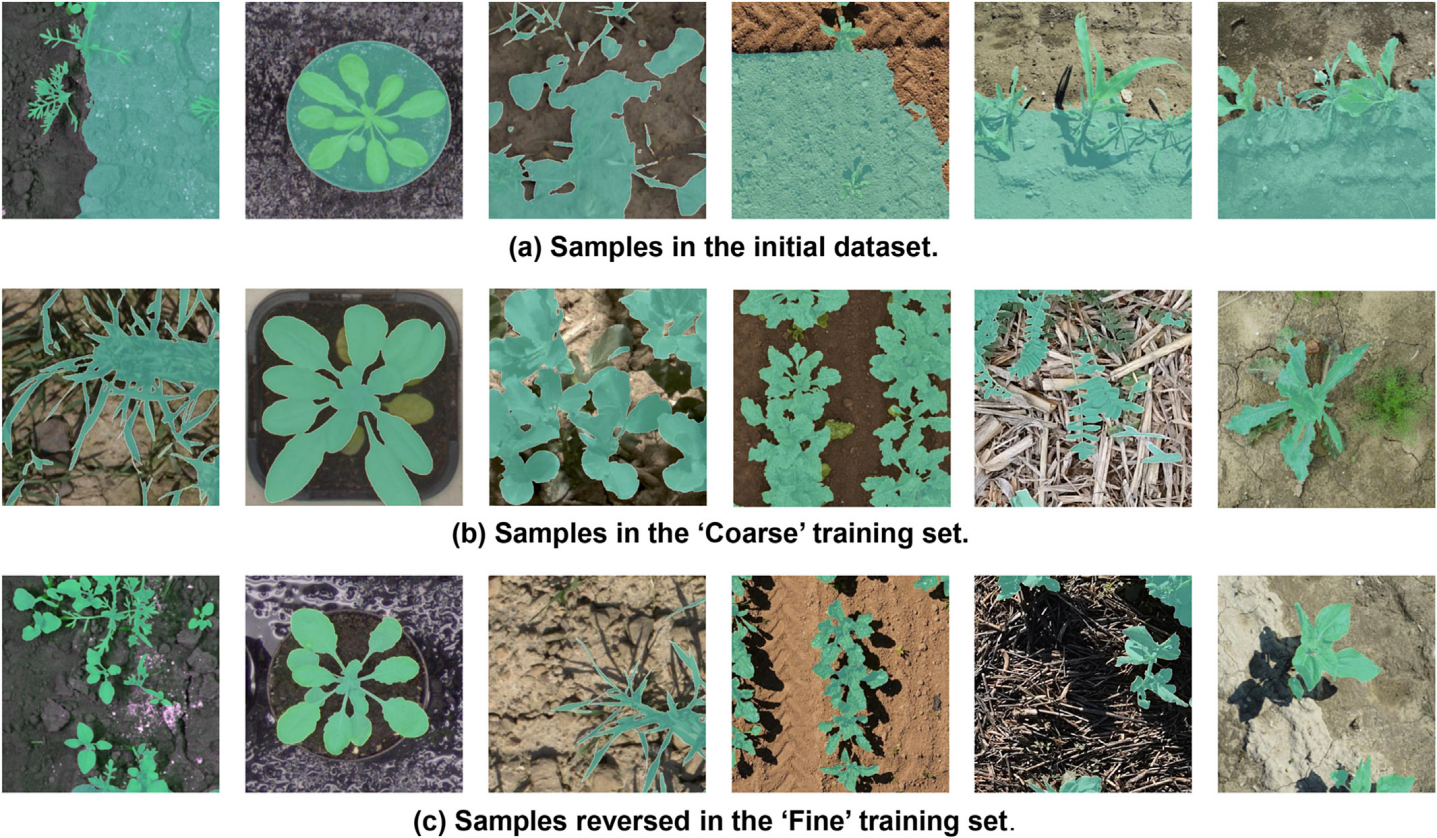
**Coarse-to-fine manual screening**. Each image is blended with its pseudo mask. Samples shown in (a) are labeled fatally wrong. They are excluded through thumbnail images during coarse screening. In this stage, we accept a certain level of mislabeling, such as the samples shown in (b). These samples survive the coarse selection but are removed during the fine selection due to incomplete foreground labels. Samples in (c) are considered to be of high quality for training.

While DAM can generate high-quality pseudo masks (Fig. 1), we observe in practice most of generations in public datasets are of low quality and cannot be directly used for training, as shown in Fig. 7(a). In fact, a prerequisite for depth-informed mask generation is that there exists a clear depth plane between foreground crop and background. Hence, in this work we manually screen the generated masks by naked eyes in a coarse-to-fine manner to filter out low-quality and obviously wrong masks. It is worth noting that, compared with pixel-level manual annotation, manual screening is rather efficient, and this is where the ‘almost unsupervised’ imply in the paper.

**Coarse Sample Selection** We first execute coarse sample selection. By blending the original RGB image with the depth-generated mask, we remove data that appear fatally wrong, e.g., a large portion of background is labeled as crops, and vice versa, as shown in Fig. 7(a). Such errors can be easily perceived from thumbnail images, so coarse screening is rather fast. The total time spent takes about only 2 ​h (40 ​min per person with three people involved). Note that, at the coarse stage, foreground crop masks with medium level of labeling errors are acceptable and retained. In all, a coarse training set of 3, 577 images are chosen from the initial 17, 199 images.

**Fine Sample Selection** Most images in the coarse training set appear acceptable initially but turns out to be problematic upon a closer inspection. Per samples shown in Fig. 7(b), some crops are only partially labeled as foreground, which can lead to frequent fluctuations of training loss. Therefore, we further filter out the coarse training set by excluding samples having missing labels for either half or all of the green crops. Such inaccurate labeling is not immediately apparent, so this process takes a little longer than the coarse selection, which takes approximately 4 ​h (2 ​h per person with two people involved). Ultimately, we streamline our training set to 1, 378 images from 3, 577 images. In all, the coarse-to-fine selection results in the refinement of our training set of 1, 378 images from 17, 199 images, and examples of the final set are shown in Fig. 7(c). The number of retained images per dataset can refer to Table S1 in the supplementary materials.

**Remark** It is worth noting that, compared with standard manual pixel *labeling by hand*, our *labeling by eyes* is significantly more efficient (normally the 6-h work may only process less than 10 crop images with hand labeling).

#### 3.5.2 Depth-informed two-stage self-training

After building the crop segmentation dataset with selected pseudo masks, one can train the crop segmentation model. However, since the pseudo masks inevitably have certain label noise, we introduce depth-informed two-stage self-training to train a robust model. Specifically, in the first stage, we train an initial crop segmentation model with the standard training pipeline. Once finished, we apply the trained model on the training set to obtain the inferred training mask ${\hat{M}}_{i}$. For the second-stage training, we generate the trimap ***M***_*c*_ conditioned on the pseudo mask ***M*** and the inferred mask ${\hat{M}}_{i}$, *i.e.*,

(9) $$M_{c}\left(i,j\right)=\left\{\begin{array}{ll} 128,\; & \text{if}\;\;M\left(i,j\right)⊕{\hat{M}}_{i}\left(i,j\right)=1\\ M\left(i,j\right),\; & \text{otherwise} \end{array}\right),$$

where ⊕ is the element-wise logical XOR operator. In ***M***_*c*_, only the confident foreground and background pixels will be identified as the second-stage training label. The uncertain regions in the trimap with a value of 128 will be ignored. This process allows us to distill the pseudo masks to improve the label accuracy. It is worth noting that, we train the second-stage crop segmentation model from the pretrained weights from the first stage. The pipeline of depth-informed two-stage self-training is summarized in Algorithm 1.

Algorithm 1 Depth-Informed Two-Stage Self-Training

**Table:** 

# DIPML: Depth-Informed Pseudo Mask Labeling, per Fig. 7(a)
# TG: Trimap Generation, per Fig. 7(b)
# f_1, f_2: stage-one and stage-two crop segmentation models
# X: crop image
# D: inferred depth map
# M: pseudo mask
# M_hat: inferred mask
# M_c: trimap mask
# Stage-One Training
# generate pseudo mask, per Fig. 6(b)
M, D ​= ​DIPML(X)
for X, M in dataloader:
M_hat ​= ​f_1(X)
loss ​= ​criterion(M_hat, M)
# Stage-Two Training
# generate trimap mask as the second-stage label, per Eqn. (9)
M_c ​= ​TG(f_1(X), D)
# inherit the stage one weights
f_2 ​= ​f_1
for X, M_c in dataloader:
M_hat ​= ​f_2(X)
loss ​= ​criterion(M_hat, M_c, ignore_label ​= ​128)

#### 3.5.3 Post-processing with depth-informed filtering

Crop segmentation models tend to generate fragile and noisy predictions. As shown in Fig. 6(d), the predicted crop masks show uneven edges and much noise. To address this, we introduce a post-processing stage with edge-preserved filtering techniques. Compared with standard image filters such as the mean filter, median filter, and Gaussian filter, edge-preserved filtering is able to filter out noise while retaining essential image details. We study two typical edge-preserved filters:

• **Guided Filter** [53] operates a target image with a weighted average conditioned on the guidance of an additional image, so it can better maintain the edge details. The radius *r* is an important parameter of Guided Filter that defines the window size of the filter. The larger the *r* is, the larger the local area would be covered by the filter, and the more pronounced the smoothing effect is, but details and edges may be blurred. A smaller *r* preserves details and edges better, but may not remove noise effectively. In the classic guided filter, the RGB image is usually used as guidance images. In our context, the refined mask ${\hat{M}}_{r}=guided\text{\_}filter\left(\hat{M},X\right)$. However, we observe in our experiments that the depth image can be a better guidance than the RGB image for mask filtering, which gives the form

(10) $${\hat{M}}_{r}=guided\text{\_}filter\left(\hat{M},D\right).$$

• **Joint Bilateral Filter** [54] is an improved version based on bilateral filter, which introduces a guidance image containing both edge and texture information to further optimize the weights, and therefore can better deal with images that lack accurate edge information. Similar to *r* in guided filter, *d* is the window diameter of the filter that controls the receptive field. *σ*_color_ is the color-space standard deviation that affects how pixel values are weighted. A large *σ*_color_ value allows more pixels of different colors to be smoothed out, while a small *σ*_color_ value limits the range of similar colors, thus preserving more color details. *σ*_space_ is the standard deviation in the spatial domain that determines how pixel positions are weighted. A large *σ*_space_ value allows the filter to consider more distant pixels, thus increasing the range of smoothing, a small *σ*_space_ value restricts the filter to only nearby pixels, which helps to better preserve image details and edges. We also use the depth image as the guidance, which amounts to

(11) $${\hat{M}}_{r}=joint\text{\_}bilateral\text{\_}filter\left(\hat{M},D\right).$$

### 3.6 Implementation and training details

**Training and Testing Data** The training data used for training the crop segmentation models are the manually screened 1, 378 images after coarse-to-fine sample selection (Section 3.5.1). The testing data uses all 5, 998 images from all the testing sets indicated in Table 1.

**Hyperparameter Setting** For gradient-guided histogram thresholding, we set the kernel size of Sobel operator to 3. The minimum threshold of Canny is 50 and the maximum threshold is 125. The gradient level *p* ​= ​3. The dilation rate used in target mask generation is 30. For post-processing filters, we iterated over the radius *r* of the Guided Filter from 1 to 15 and over the *d* of the Joint Bilateral Filter from 1 to 200 incrementally, with *σ*_color_ ​= ​25 and *σ*_space_ ​= ​15.

**Experimental Platform** All experiments are conducted on the Ubuntu 20.04 system with Python version 3.8, PyTorch version 1.10.0, CUDA version 11.3, and on a workstation with eight 24 ​GB RTX3090 GPUs, two 10-core Intel Xeon Silver 4210R CPUs, and 256 ​GB RAM.

**Training Setting** The size of the input image is cropped to 256 ​× ​256, we train the network with the Stochastic Gradient Descent (SGD) optimizer with the momentum of 0.9, weight decay of 0.0005, initial learning rate of 1*e* ​− ​3, minimum learning rate of 1*e* ​− ​4. The batch size is 8, and the total number of iterations is 15, 000. The loss function uses the cross-entropy loss.

## 4 Results

In this section, we first introduce the baselines, experimental protocols, and evaluation metrics. We then conduct a series of ablation studies to validate each component of DepthCropSeg.

### 4.1 Baselines, experimental protocols, and evaluation metrics

**Baselines** We choose four semantic segmentation models as the base crop segmentation models. Among them, DeepLabV3+, SegFormer, and Mask2Former incorporate the design of multi-scale feature fusion:

• **U-Net** [27]: Based on an encoder-decoder architecture, it features a U-shaped, symmetric structure with multiple up-sampling and down-sampling stages, suitable for crop segmentation.
• **DeepLabV3+** [28]: With an architecture consisting of atrous convolution in the encoder for context capture and a decoder module for boundary refinement, it offers advantages in capturing fine details and multi-scale context, especially at object boundaries.
• **SegFormer** [29]: It innovates with a position-free hierarchical encoder and an efficient MLP decoder, simplifying segmentation for high efficiency and accuracy.
• **Mask2Former** [30]: It introduces a universal segmentation architecture with masked attention for localized feature extraction, achieving state-of-the-art performance.

In addition to the standard segmentation baselines above, we also evaluate the performance of different mask generation approaches:

• **Depth-OTSU**: It directly generates the crop mask by binarizing the depth map with OTSU thresholding.
• **Depth-GHT**: It uses Bayesian methods to analyze histograms to determine the optimal threshold before binarizing the depth map to generate the crop mask.
• **Depth-Ours**: This is our proposed mask generation approach. We use this baseline to assess the quality of generated mask.

Further, we take two state-of-the-art category-agnostic segmentation models into comparisons:

• **SAM** [22]: SAM is a recent category-agnostic segmentation model featuring a flexible prompt encoder and a lightweight mask decoder. It leverages a powerful image encoder to generate image embeddings, which are then used in conjunction with prompts such as points, boxes, or masks to produce accurate segmentation masks in real-time. SAM excels in general segmentation tasks, enabling zero-shot transfer to new data distributions and downstream tasks via prompt engineering.
• **HQ-SAM** [31]: It enhances the original SAM by introducing a high-quality output token and global-local feature fusion, designed to improve the segmentation quality without compromising the zero-shot capabilities of SAM. It achieves superior mask quality by refining features from the Vision Transformer (ViT) encoder and by integrating them with mask decoder features, ensuring detailed and precise segmentation.

**Experimental Protocols** We train four semantic segmentation baselines on our coarse training set and on our fine training set, respectively. For comparison, we also conduct training on the subset with exactly the same samples above, but using manual annotations. The U-Net network adopts a randomly initialized ResNet-101 backbone, DeepLabV3+ uses ImageNet-pretrained ResNet-101, Mask2Former uses ImageNet-pretrained Swin-T, and the B5 version of SegFormer is used. The two-stage training is only applied to the best-performing model above, followed by post-processing filtering. For SAM and HQ-SAM, we randomly select one to ten points from the ground-truth crop masks as prompts and retain the result with the highest mIoU.

**Evaluation Metrics** We use the mean intersection over union (mIoU) over the entire testing data to evaluate the quality of the masks, which is computed by the mean ratio of the area of overlap to the area of union and defined by

(12) $$\text{mIoU}=\frac{1}{C}\overset{C}{\underset{c=1}{\sum }}\frac{\sum_{n=1}^{N}M^{n}\left(c\right)\cap M_{\text{gt}}^{n}\left(c\right)}{\sum_{n=1}^{N}M^{n}\left(c\right)\cup M_{\text{gt}}^{n}\left(c\right)},$$

where *C* is the number of classes, and *N* is the number of testing data. In this paper, *C* ​= ​2 because crop segmentation is simply a foreground-background problem. Given the strong class imbalance and significant variations in the amount of data selected from different datasets in our combined dataset, we also compute and report the mIoU score for each individual dataset and the overall mIoU for a comprehensive assessment of performance. We use the code provided by mmsegmentation.^3^

### 4.2 Comparison of different crop segmentation models

**Fig. 8:**
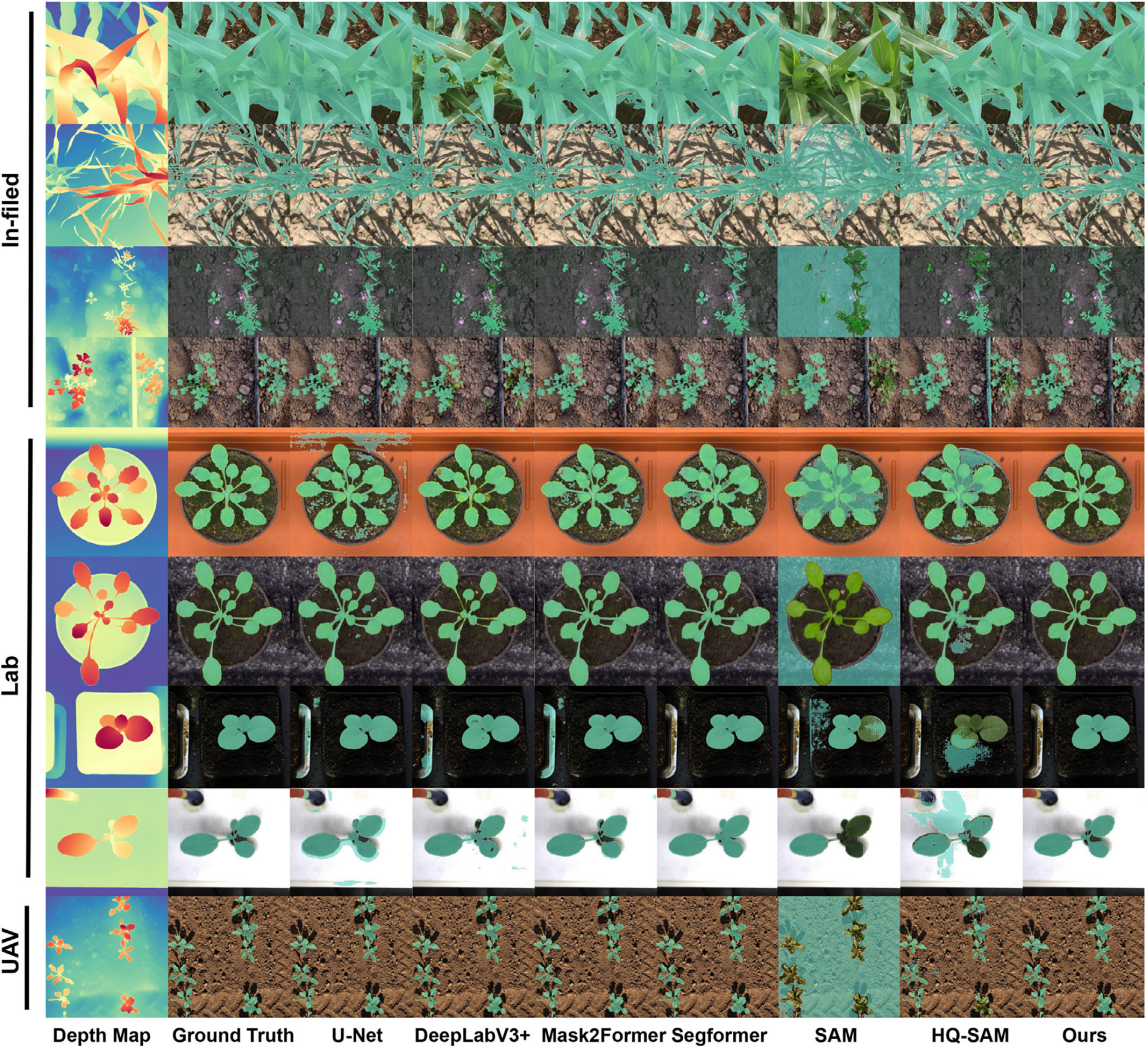
**Comparison of fully supervised and semi-supervised segmentation approaches**. From top to bottom, the figure illustrates pseudo-colored masks for eight crop images across in-field, Lab, and UAV scenarios. From left to right, it presents depth maps, GT, and results from both fully supervised and semi-supervised approaches.

Results of different crop segmentation approaches on the ten public datasets are shown in Table 2 and Fig. 8. As aforementioned in Section 4.1, four semantic segmentation models that employ the ground truths as the training masks are indicated as ‘full supervision’. Correspondingly, DepthCropSeg refers to the models employing masks generated by our proposed mask generation approach (Depth-Ours). According to Table 2, since SegFormer exhibits the best overall performance among the four models, we proceed to train this model with depth-informed two-stage self-training, which is indicated with an asterisk (∗). From Table 2, one can observe that: i) the performances of DepthCropSeg with different base models are comparable to the full-supervision ones, less than 0.2 mIoU; ii) DepthCropSeg has demonstrated a significant improvement over the direct mask generation from depth models (a 10 ​+ ​mIoU improvement) and over the Segmentation Anything models (an approximate +20 mIoU boost); iii) performances of different base models vary significantly across different datasets, suggesting data distributions and base segmentation models matter. Qualitative comparison of different approaches is shown in Fig. 5. Our DepthCropSeg produces the most visually pleasing segmentation masks with smooth regions and sharp boundaries.

**Table 2:** Performance comparison of different approaches. ∗ means the model trained with depth-informed two-stage self-training. Best performance under ‘full supervision’ is underlined, and that with DepthCropSeg is in **boldface**.

Supervision	Base Model	CWFID	CVPPP	EWS	PhenoBench	VegAnn	CNW	MSUPID	KOMATSUNA	Carrot-Weed	HUST-Crop	Overall
No Supervision	Depth-OTSU	46.36	35.35	54.88	54.91	47.61	30.91	62.49	42.08	64.84	45.18	36.04
	Depth-GHT	66.37	36.64	55.39	65.98	53.51	27.12	64.07	38.58	81.09	47.17	34.07
	Depth-Ours	83.86	50.99	72.99	90.90	58.14	83.16	69.47	87.69	75.12	70.55	76.16
	SAM	27.36	53.81	36.34	28.12	50.55	35.70	64.40	52.96	35.19	41.98	38.19
	HQ-SAM	55.49	64.33	50.59	68.52	59.35	62.24	65.23	58.12	62.35	61.15	67.41
Full Supervision	UNet	77.99	77.77	81.07	92.87	67.36	88.04	37.58	84.68	49.82	87.45	82.97
	DeepLapV3+	79.60	92.82	80.23	90.60	69.37	87.50	28.73	84.09	51.54	71.62	83.80
	Mask2Former	86.23	64.78	85.18	93.91	67.65	87.99	50.27	85.24	73.95	85.84	82.76
	SegFormer	88.33	83.34	84.86	93.92	69.52	89.65	40.48	90.12	73.82	82.96	87.10
DepthCropSeg	UNet	73.12	78.18	80.34	91.26	**67.68**	84.12	36.69	86.42	50.40	82.59	81.59
	DeepLapV3+	81.59	79.38	74.18	87.92	55.39	79.53	60.35	80.37	53.10	63.21	77.58
	Mask2Former	88.06	72.04	**83.90**	**92.96**	67.65	**88.44**	49.30	54.65	75.98	**86.67**	83.45
	SegFormer	**89.89**	77.60	82.67	92.38	64.99	87.95	**73.31**	83.90	74.68	85.69	85.21
	SegFormer∗	89.78	**91.04**	80.51	92.85	59.63	88.05	71.27	**89.71**	**76.62**	81.79	**86.91**

### 4.3 Ablation studies

Here we conduct a series of ablation studies to justify each of our design choices.

**Effectiveness of Manual Screening** Here we investigate the effectiveness of manual screening on the depth-generated training set. To validate the necessity of fine sample selection stage, we train two models on the ‘fine’ training set with pseudo labels and on the same samples with ground-truth manual labels, respectively. The performance of various segmentation baselines on the ‘coarse’ and ‘fine’ training sets are shown in Table 3a. It can be found that models trained on the ‘fine’ set achieves consistently better performance than that trained on the ‘coarse’ set, with +2 ∼ + 5 mIoU improvements.

**Table 3:** Ablation study of DepthCropSeg.

(a) Effectiveness of the ‘coarse’ and the ‘fine’ training set.		
Model	Coarse	Fine
U-Net	78.56	81.59
DeepLabV3+	75.82	77.58
Mask2former	80.25	83.45
SegFormer	80.19	85.21
SegFormer∗	81.05	86.91

(b) Effectiveness of different post-processing filters under different guidance.		
Guidance	Guided Filter	Joint Bilateral Filter
No Post-process	86.91	
RGB	86.96 (+0.05)	87.19 (+0.28)
Depth	87.17 (+0.26)	87.23 (+0.32)

(c) Effectiveness of each design choice in DepthCropSeg with SegFormer as the base segmentation model. The first row is the result of Depth-OTSU, which could coarsely represent the lower bound.					
Depth-informed mask labeling	Coarse selection	Fine selection	Depth-informed two-stage training	Depth-informed post-processing	mIoU
					36.04
✓					76.16
✓	✓				80.19
✓	✓	✓			85.21
✓	✓	✓	✓		86.91
✓	✓	✓	✓	✓	87.23
Full supervision					87.10
Full supervision w. post-processing					87.12

**Fig. 9:**
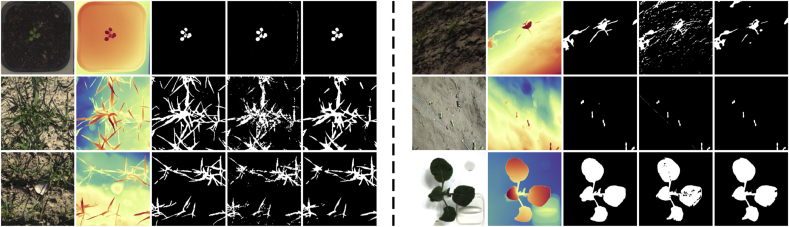
**Visualizations of the Joint Bilateral Filtering results**. For each each set of visualizations, from left to right, the RGB image, the depth image, the ground truth, the result of SegFormer∗, and the result after Joint Bilateral Filter.

**Effectiveness of Depth-Informed Self-Training and Filtering** Here we investigate the impact of depth-informed self-training and depth-informed filtering. The results of the former are shown in the last row of Table 2. Depth-informed self-training lifts the performance from 85.21 to 86.91. To see whether incorporating depth information or the RGB information into post-processing filtering, we compare two edge-preserved filtering baselines, *i.e.*, Guided Filter (GF) and Joint Bilateral Filter (JBF), and alternate the guidance image between the depth map and the RGB image. Quantitative and qualitative results are shown in Table 3b and Fig. 9, respectively. We remark that, while the numerical improvements seem not significant (around +0.2 mIoU) due to minor changes of region predictions, the denoising effect of the edge-preserved filtering is obvious from the qualitative illustrations.

**Fig. 10:**
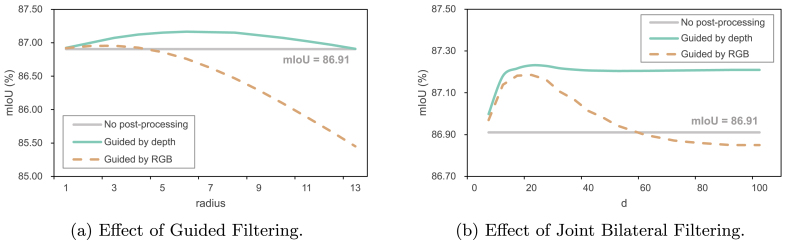
**Performance curves of two post-processing filters with different guidance**. The baseline used is the SegFormer∗ without post-processing whose mIoU is 86.91.

**Parameter Sensitivity of Depth-Informed Filtering** While post-filtering has proven to be effective, it has certain hyperparameters. Here we further examine the parameter sensitivity used in different filters. In particular, we iterate over a range of values of the radius *r* of GF and of the diameter *d* of JBF to see whether performance is sensitive to a specific parameter chosen. Other parameters are kept unchanged for fair comparison. Results are shown in Fig. 10. Filtering guided by depth behaves better than filtering guided by RGB, and depth guidance is also insensitive to hyperparameter change. In contrast, RGB guidance shows a negative effect on the mIoU once the parameter is outside the appropriate configuration (*r* ​> ​5 for GF and *d* ​> ​60 for JBF). According to the results, we finally choose the JBF as our post-processing filter, with depth guidance. Note that using a JBF with a large *d* can significantly increase computational time. Thus we recommend setting *d* ​= ​20.

**Accumulated Effectiveness of Each Component** Here we provide an overview on each contribution of different components in our approach. As shown in Table 3c, we report the performance on the testing set after integrating additional components over the SegFormer baseline, by order. We also include two fully supervised baselines trained using ground truth annotations. For fairness, we also post-process the full-supervision results with the same depth-informed filter. It can be seen that DepthCropSeg achieves the best performance of 87.23 with all components included. Note that our best result is even slightly better than the full-supervision baseline.

### 4.4 Qualitative comparison on self-collected data

**Fig. 11:**
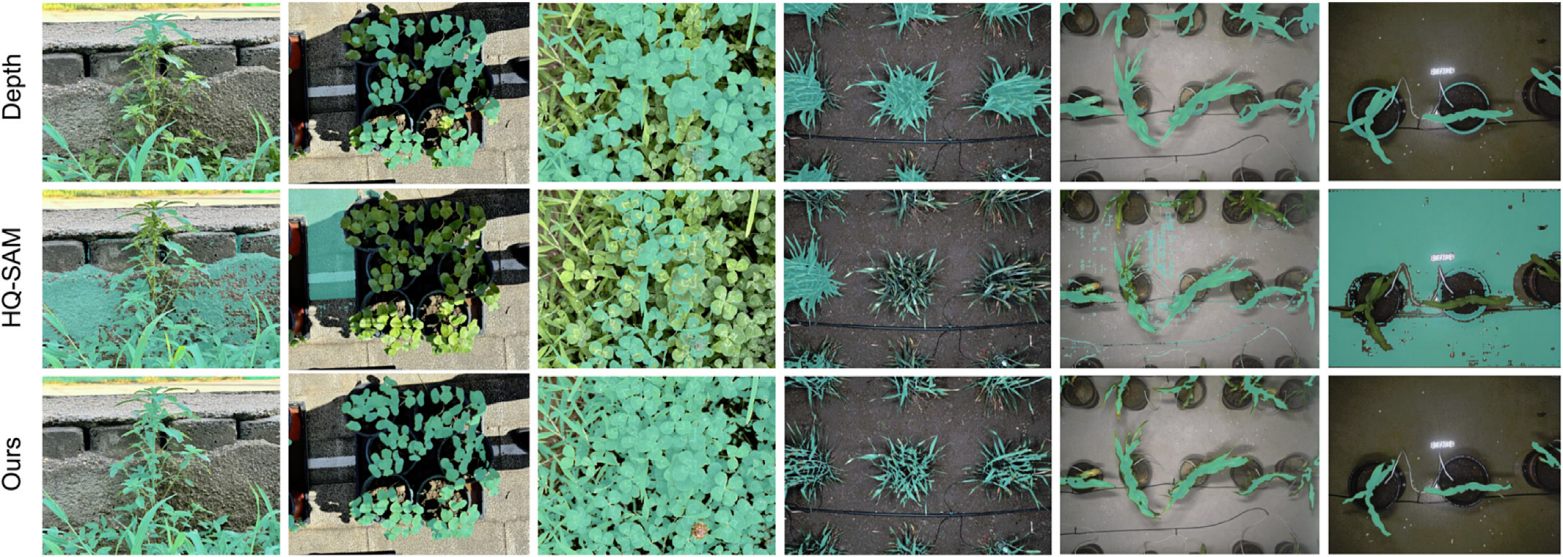
**Visualizations on images collected by TraitDiscover and captured by smartphones**. DepthCropSeg remains effective on self-collected data with different distributions. Additional visualizations could be found in Fig. S1 and Fig. S2 of the supplementary materials.

Since we do not provide manual annotations for self-collected data, here we mainly provide a qualitative comparison between Depth-Ours, HQ-SAM, and DepthCropSeg, which may be sufficient to demonstrate the efficacy of DepthCropSeg. In Fig. 11, we visualize predictions on images collected by TraitDiscover and by phone. By viewing different samples, one can find that, even if some foreground points are manually chosen as prompts for HQ-SAM, HQ-SAM still incorrectly segments a significant portion of the background as foreground. When plants almost completely occupy the image (the second column), the masks generated by Depth-Ours and HQ-SAM show considerable under-segmentation. For laboratory scenarios, our DepthCropSeg also performs better than the other two in segmenting plants from pots.

### 4.5 Failure cases

**Fig. 12:**
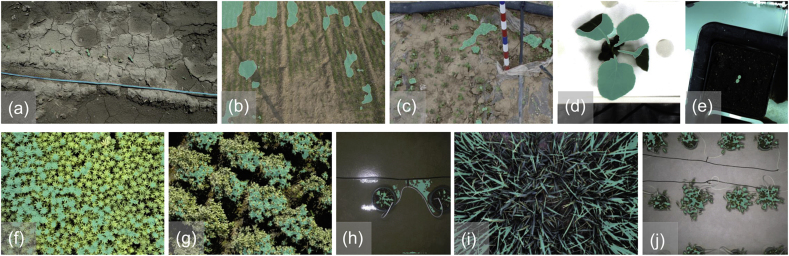
Some failure cases of DepthCropSeg.

Here we show some failure cases of DepthCropSeg. Sometimes DepthCropSeg can fail to achieve accurate segmentation. In Fig. 12, we plot the segmentation masks predicted by DepthCropSeg. We see some typical failure patterns. e The method can fail when the depth difference between the plants and the background is marginal (Fig. 12(a–c), or when the plants densely occupy the entire image (Fig. 12(f)(g)(i)). ii) Aerial imaging can lead to partial detection of the plants and may mistakenly identify objects that do not appear during training such as the pipes. iii) Additionally, in some laboratory images, if the leaves are curled, the surrounding area may not be appropriately segmented (Fig. 12(d)). iv) In some potted plants where the plants are extremely small (Fig. 12(e)), misclassification can also occur. Some failure examples of images captured by TraitDiscover and smart-phones is illustrated as Fig. 12(f) (j). Such failure cases may be mitigated with additional training data and improved segmentation models.

**Fig. 13:**
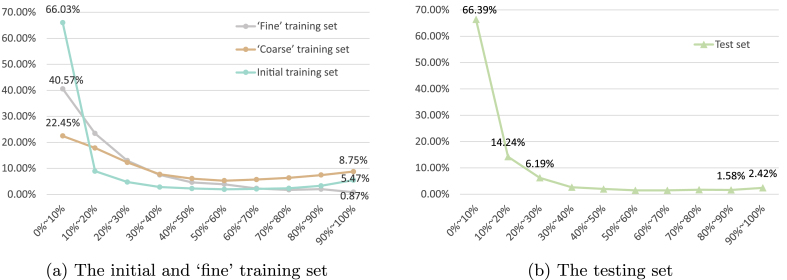
**Comparison of coverage distribution of the initial training set, ‘coarse’ training set, ‘fine’ training set, and testing set**. The number represents the proportion of the crop area to the entire image. Depth-informed pseudo labeling and manual screening may favor crop images of less canopy coverage and potentially lead to a somehow biased training set.

The limitation i) above indicates that DepthCropSeg may struggle in scenarios with full or near-full canopy coverage. To explain this, we visualize the canopy coverage distribution across the training and test sets (Fig. 13). It reveals that, in the manually filtered ‘Fine’ training set, the number of images with less than 20 ​% canopy coverage is approximately 22 times greater than those with over 80 ​% coverage. In contrast, the original training set shows a ratio of only 8.6. This indicates that manual screening may be biased toward images with lower canopy coverage. Fortunately, it seems easy to acquire masks of full-canopy images to augment the training set.

## 5 Discussions

**Depth Anything Model emerges to be a tool for high-quality pseudo labeling of crop masks**. In the realm of crop segmentation, classic pixel-level annotation methods are laborious and costly. To address this challenge, numerous approaches have been developed to generate pseudo masks as a substitute for manually annotated data. However, these approaches have the disadvantage of requiring additional computational resources (*e.g.*, self-supervised learning often needs large-scale pretraining) and may achieve the sub-optimal segmentation accuracy. In the context of foundation models, the strong generalization capability of these models makes it possible to generate zero-shot segmentation masks. The SAM model, proposed in 2023, is the most significant achievement in the field of image segmentation recently. But it should be noted that SAM is trained on natural images and is difficult to behave well on agricultural data with complex and variable backgrounds. Although improved models like HQ-SAM have shown enhanced detail delineation, they still struggle with the robustness against lighting and shadows in the agriculture domain. Given the clear depth difference between plants and soil in field-based images, it seems easy to think about obtaining segmentation masks from the depth map. However, before the emergence of Depth Anything V2, the quality of depth maps is poor (even the V1 model cannot be used to generate accurate crop masks). This leads to a sense of generating pseudo masks from depth is difficult to implement. However, as shown in Fig. 4, the quality of the depth map generated by Depth Anything V2 is remarkably superior to those produced by Depth Anything V1. We may just reach a timestamp that depth could be useful for plants. Indeed our experiments demonstrate that this model is robust to variations in illumination, shadows and cluttered background. It is also capable of discerning the intricate details of leaf edges, even on crop instances where two leaves are partially occluded. In a word, Depth Anything V2 emerges to be a powerful tool for generating high-quality pseudo labels for crop masks.

**Depth-informed pseudo labeling achieves close-to-full-supervision crop segmentation performance**. A rather inspiring result of this paper is that DepthCropSeg achieves close-to-full-supervision crop segmentation performance (Table 2), suggesting the quality of depth-generated masks is similar to the manual annotations. Although the depth-generated masks still contain some noise after coarse-to-fine manual screening (Section 3.5.1), it has little impact on the learning of the model. It is worth noting that DepthCropSeg occasionally reports better results than the models trained with manually labeled masks, the Mask2Former model for instance. This suggests manual annotations sometimes can be erroneous and may not be the optimal supervision signal. The model with the best overall performance is not all the best on each dataset. This may be owing to different image resolutions of each dataset, and different models have different adaptations to resolution.

**Manual screening and supervised training remain essential**. According to Table 2, although the quality of masks generated by Depth-Ours on each dataset is generally better than that of Depth-OTSU and Depth-GHT, all three methods fall significantly short of meeting the requirements for crop segmentation tasks. On the other hand, category-agnostic segmentation can be achieved without further supervised training, as seen in models like SAM and HQ-SAM. However, the segmentation performance of these models also falls below 70 ​% mIoU, which is insufficient to meet practical needs. In contrast, in DepthCropSeg, the segmentation performance of the semantic segmentation models is much better than that of the two state-of-the-art category-agnostic ones. This suggests that manual screening and supervised training remain indispensable.

**Manual screening is a time-efficient way to provide manual supervision**. Different from common practices in weakly supervised learning, human supervision is used in a simple and time-efficient way in this work. Manual screening at the coarse sample selection can be considered providing image-level labels, but at the fine stage the screening actually provide pixel-level annotations by eyes, instead of by hands. We remark that, compared with pixel-level annotations by hand, coarse-to-fine manual screening is significantly more efficient. In Section 3.5.1, we have discussed the time spent filtering our training dataset. Our coarse-to-fine selection takes around 6 ​h in total and finally results in a coarse training set of 3, 577 images and a fine training set of 1, 378 images. Taking manual pixel-level annotations mentioned in the labeling process of PhenoBench [41] as an instance, the team works approximately 2, 000 ​h to obtain a high-quality dataset of 2, 872 images, where crops, weeds, and leaves are labeled, respectively. Compared with the time cost of manual labeling of **42 ​min** per image in PhenoBench, we cost only **15 ​s** on average to obtain a pseudo mask for an image in our ‘Fine’ training set. While we do not differentiate between crops and weeds during segmentation, the time cost for additional relabeling the crops and weeds would still be within an acceptable range (no pixel-annotation is required).

**Manual screening stage may be biased toward less mature plants**. As illustrated by the failure cases in Fig. 12(f)(g)(i), DepthCropSeg does not perform well in scenarios with full or near-full canopy coverage. In Fig. 13, it could be observed that the proportion of images with over 80 ​% coverage in the ’Fine’ training set is significantly reduced after fine screening. This is due to the reduced distinction between the ground plane and the plants in such scenarios, making depth perception ambiguous. Consequently, a number of high-coverage samples were lost during data screening, leading to a low proportion of such samples in the final training set. As a result, the segmentation model struggles to learn the features of high-coverage samples. In the testing set we collected, the percentage of full-coverage plants in the whole dataset is also relatively low, so the segmentation performance on the testing set can still be good enough. It must be acknowledged that full canopy coverage (close to 100 ​%) is common in many field crops. Therefore, when using DepthCropSeg in reality, additional full or near-full canopy coverage samples should be supplemented into the training set. If this proportion is too low, manual adjustments should be considered to mitigate the excessive exclusion of such samples during model training. Fortunately, acquiring labels for full-coverage images seems easy.

**Ways for generating pseudo masks matter**. We have studied three different ways for generating pseudo masks from depth maps. As shown in Fig. 5, the classic thresholding approaches do not perform well on the depth maps with biased background. This means the number of candidate data to be screened would be significantly reduced if one uses classic image thresholding. Targeting this dilemma, we develop a specialized approach to generate pseudo masks from varied depth maps. This approach can significantly enrich the candidate training pool. The first three rows in Table 2 also confirm the effectiveness of our proposition, achieving an overall mIoU of 76.16.

**Mask quality outweighs mask quantity**. In deep learning, there is a common sense that training a deep model requires a large number of training data. In Section 3.5.1, we apply the fine sample selection to improve the mask quality, but significantly reduce the number of training images from 3, 577 to 1, 378. A question naturally arises: Mask quality or mask quantity, which is more important? According to Table 3a, segmentation models trained on the ‘Fine’ set achieve consistently better performance than that trained on the ‘Coarse’ set. Therefore, at least for crop segmentation, mask quality seems more important than mask quantity when the difference of sample size is no more than an order of magnitude.

**Crop segmentation masks can be further refined by post-using depth information**. Despite the favorable performances of crop segmentation baselines trained on pseudo labeled masks (Table 2), there exists much noise in the model predictions. For example, the model may have significant salt-and-pepper noise, incorrectly mismatch parts of foreground, or misinterpret background (Fig. 9). To address this, we apply post-processing with edge-preserved filtering, which has proven to effectively remove noise while preserving edge details. From Fig. 10, it is clear that post-filtering works. In contrast to the standard practice that uses RGB guidance, we find that depth guidance works better. A possible reason is that the depth map prefilters unimportant information such as illumination and background. It is noteworthy that applying the post-processing filter to the fully supervised model which is considered as our higher bound ​+ ​yields only marginal improvement and does not surpass the performance of DepthCropSeg with post-filtering (Table 3c). A plausible explanation is that depth-guided filtering more effectively assists deep-pseudo-masks-based models in eliminating noise, as it possibly leverages additional information or corrects systematic biases introduced during the training process.

**DepthCropSeg perhaps indicates a new learning paradigm for crop segmentation**. In this work, we claim that DepthCropSeg is an *almost unsupervised* approach to crop segmentation. Here we clarify that we claim this because we think the learning of DepthCropSeg does not belong to any existing learning paradigms. Compared with standard fully-supervised learning in segmentation, DepthCropSeg does not exploit the conventional hand-labeled crop masks. It also falls into neither the domain of semi-supervised learning nor weakly-supervised learning, because DepthCropSeg does not rely on weak labels or unlabeled data as well. The core of DepthCropSeg lies in its minimal reliance on human intervention; it replaces labor-intensive hand annotation with fast visual screening, significantly reducing the cost of human effort in data labeling. We believe this new almost unsupervised learning paradigm introduces a groundbreaking framework for crop segmentation, offering a cost-effective and scalable alternative for efficient label creation.

## 6 Conclusion

In this work, we show the powerful potential of Depth Anything Model (DAM) in generating high-quality pseudo crop segmentation masks, thus evading the need of manual pixel-level annotations in deep-learning based approaches. With the blessing of DAM, we introduce a depth-informed crop segmentation approach, DepthCropSeg, to the plant phenotyping community, which enables almost unsupervised crop segmentation. We benchmark the segmentation performance of this approach on ten public crop datasets including field-based, laboratory, and UAV scenarios and report performance close to the model trained with manually labeled masks. We also conduct a number of ablation studies to justify each of our design choices. In addition, we further verify the generalization of DepthCropSeg on self-collected data. The results show that our DepthCropSeg achieves close-to-full-supervision performance, maintains strong robustness, while requiring almost no manual labeling.

One limitation of the current approach is the bias introduced by the manual screening stage, which may excessively reduce the number of samples with full canopy coverage. We plan to incorporate additional samples with full canopy coverage into the training process, as obtaining masks for such samples appears to be relatively low-cost. Such an enhancement will allow us to build a self-iterative data engine that improves the success rate of manual screening and enables more data to participate in model training. Ultimately, we aim to create a universal crop segmentation foundation model capable of tackling various scenarios effectively, while significantly reducing the reliance on manual annotation in data preparation.

## Author contributions

S. Cao conceived the idea, implemented the method, designed and conducted the experiments, collected and validated the data, and drafted and revised the manuscript.

B. Xu conducted the experiments, collected and managed the dataset, conducted the data visualizations, and drafted and revised the manuscript.

W. Zhou conducted the experiments, collected and managed the dataset, conducted data analysis, and drafted and revised the manuscript.

L. Zhou conducted the experiments, contributed to data curation, helped in data analysis, and drafted and revised the manuscript.

J. Zhang managed the project, participated in experiments and discussions, and reviewed and revised the manuscript.

Y. Zheng investigated specific aspects of the methodology and reviewed the manuscript.

W. Hu co-supervised the project, provided the funding and data, and reviewed and revised the manuscript.

Z. Han co-supervised the project, provided the funding and data, and reviewed and revised the manuscript.

H. Lu is the project lead, conceived the idea, developed the technical route, coordinated the experiments, and reviewed and revised the manuscript.

## Data availability

The crop segmentation datasets used by this study are publicly available. The self-collected data are also available upon request.

## Funding

This work was jointly supported by the 10.13039/501100002367Chinese Academy of Sciences “Strategic Priority Research Program” Under Grant No. XDA24040201, 10.13039/100006180Central Government's Guidance Fund for Local Science and Technology Development Under Grant No. 2024ZY-CGZY-19, 10.13039/501100001809National Natural Science Foundation of China Under Grant No. 32370435, National Key R & D Program of China Under Grant No. 2023YFF1001502, and Changchun Science and Technology Development Programme Under Grant No. 23SH18.

## Declaration of competing interest

The authors declare that they have no known competing financial interests or personal relationships that could have appeared to influence the work reported in this paper.

## Footnotes

2 In this paper, we slightly abuse the term ‘crop’ as we do not discriminate crop and weed and consider general greenness extraction.

3 https://github.com/open-mmlab/mmsegmentation.
