# Supplementary material for: The blessing of Depth Anything: An almost unsupervised approach to crop segmentation with depth-informed pseudo labeling
Source: Plant Phenomics. 2025 Feb 27;7(1):100005. doi: 10.1016/j.plaphe.2025.100005 (PMC12709960; doi:10.1016/j.plaphe.2025.100005)
Supplement: Multimedia component 1 [file mmc1.pdf]

Supplementary Materials

We include additional figure illustrations in the supplementary:

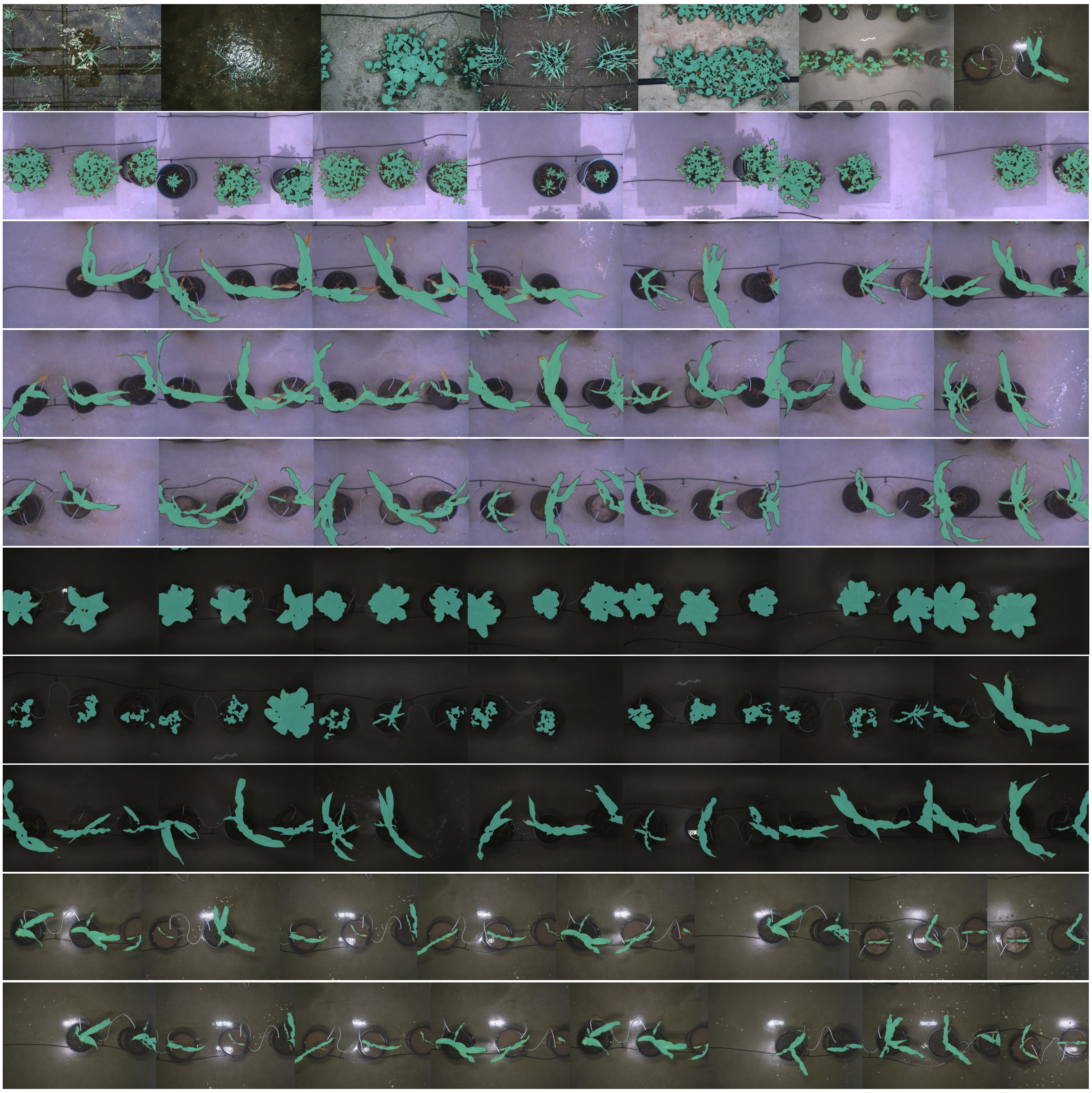

Figure S1: **Visualizations of the segmentation mask generated by DepthCropSeg on images collected by TraitDiscover**.

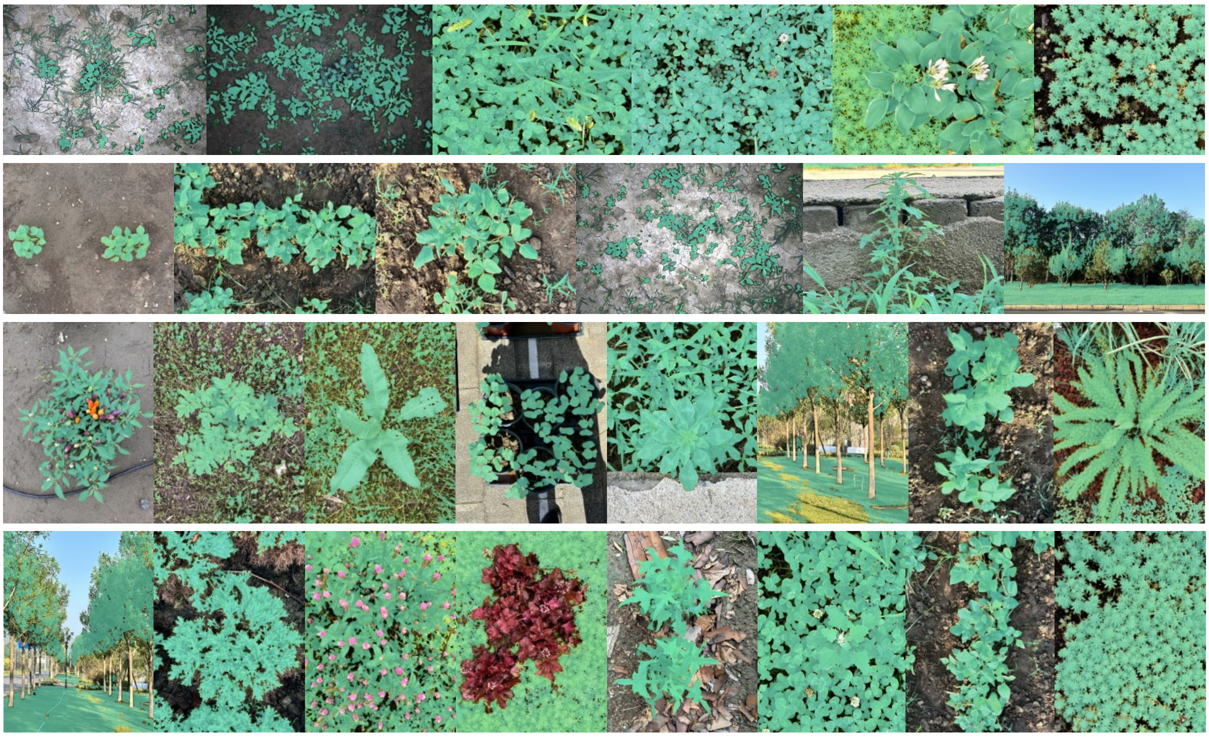

Figure S2: **Visualizations of the segmentation mask generated by DepthCropSeg on images captured by smart-phones.**

We include the number of retained images in each training dataset after coarse-to-fine manual screening in the supplementary:

Table S1: Number of retained images for training after coarse-to-fine manual screening.

Dataset	#Images	#Coarse Selection	#Fine Selection
CWFID	60	8	5
CVPPP	1,311	403	313
EWS	190	47	7
PhenoBench	2,179	1,024	558
VegAnn	3,775	1,790	337
Crop And Weed	8,034	305	118
Overall	17,199	3,577	1,378
